# Iron, Emotion, and Awareness: Exploring Alexithymia and Anxiety in Anemic Women

**DOI:** 10.3390/medicina61081359

**Published:** 2025-07-26

**Authors:** Bercem Afsar Karatepe, Sevler Yıldız, Tuğçe Taşar Yıldırım

**Affiliations:** 1 Department of Internal Medicine, Elazığ Fethi Sekin City Hospital, Elazığ 23300, Turkey; ttasar_09@hotmail.com; 2 Department of Psychiatry, Elazığ Fethi Sekin City Hospital, Elazığ 23300, Turkey; dr_sevler@hotmail.com

**Keywords:** iron-deficiency anemia, women’s health, alexithymia, anxiety disorders, depressive disorder, quality of life, emotional processing, mental health, hematologic diseases, cross-sectional studies

## Abstract

Despite being highly prevalent among women of reproductive age, the psychological dimensions of iron deficiency anemia (IDA) often go unrecognized. While the hematological consequences of IDA are well established, emerging evidence suggests that it may also adversely affect emotional processing, mental health, and overall quality of life. This study aimed to systematically assess levels of alexithymia, anxiety, depressive symptoms, and quality of life in women diagnosed with IDA compared to age-matched healthy controls. A total of 151 women with confirmed IDA and 150 healthy controls were recruited. Participants underwent laboratory testing and completed validated questionnaires, including the Beck Depression Scale (BDS), State-Trait Anxiety Inventory (STAI), WHOQOL-BREF-TR, and the Toronto Alexithymia Scale (TAS-20). Women with IDA demonstrated significantly higher alexithymia and anxiety scores and lower quality of life compared to controls. Within the IDA group, probable alexithymia was associated with more severe anemia parameters and poorer psychological outcomes. These findings indicate that IDA is not only a hematological disorder but also one with a substantial psychological burden. Recognizing and addressing these psychological dimensions in clinical practice is critical. A multidisciplinary management approach that integrates both hematological treatment and mental health interventions may be essential to improve overall patient outcomes among women with IDA.

## 1. Introduction

Iron deficiency anemia (IDA) is one of the most common nutritional illnesses globally, significantly affecting women of reproductive age. The World Health Organization (WHO) reports that over one-third of women worldwide are affected by anemia, primarily due to iron deficiency [1]. This burden is particularly evident in low- and middle-income nations, where nutritional deficits, infections, and restricted healthcare access converge to heighten vulnerability [2]. In addition to serving as a marker of systemic health inequities, IDA presents a considerable clinical problem owing to its varied and frequently unacknowledged health ramifications.

Traditionally, IDA has been primarily addressed as a hematological disorder, with strategies focused on replenishing iron reserves and rectifying anemia via dietary adjustments, oral supplements, or intravenous iron therapy. These treatments effectively normalize hemoglobin levels and mitigate characteristic symptoms, such as fatigue and weakness. Emerging data suggest that iron’s biological roles extend beyond erythropoiesis, implicating it in many neurophysiological processes crucial for brain function and emotional regulation [3,4,5].

Iron is an essential component in the production and metabolism of monoamine neurotransmitters, such as dopamine, serotonin, and norepinephrine, which govern mood, cognition, and executive function [3,4]. Experimental and clinical studies have shown that iron shortage can disturb neurotransmitter equilibrium, hinder oligodendrocyte function, and diminish myelin synthesis, resulting in structural alterations in white matter tracts essential for emotional processing and interregional communication [5,6]. These alterations impact brain regions such as the hippocampus, basal ganglia, anterior cingulate cortex, and insula, which are crucial to emotional awareness and control. Functional neuroimaging studies validate these neuronal changes by demonstrating abnormal activation patterns in these regions among people with iron insufficiency [7].

Furthermore, iron deficiency results in cerebral hypoxia, mitochondrial dysfunction, and low-grade systemic inflammation, which all impair cellular energy metabolism and immunological signaling pathways pertinent to emotional regulation [8,9]. The neurochemical, structural, and metabolic mechanisms collectively offer a credible biological basis for the identified correlations between iron deficiency and heightened susceptibility to psychiatric disorders, such as depression, anxiety disorders, sleep disturbances, and psychotic symptoms [6,7].

The acknowledgement of these neurological processes has redefined iron deficiency as a multifaceted systemic illness with considerable neuropsychiatric consequences. Evidence indicates that addressing iron deficiency may enhance anxiety and depressive symptoms, underscoring the possible reversibility of these psychiatric manifestations through suitable therapies [8,9]. Nonetheless, the majority of current research has concentrated on general psychiatric outcomes, failing to adequately examine specific emotional regulation challenges that could influence these relationships.

Alexithymia is a personality trait characterized by challenges in recognizing and articulating emotions, coupled with an externally orientated cognitive style. Alexithymia has been linked to several mental and neurological illnesses, such as depression, anxiety, substance use disorders, and somatoform problems [10,11]. Its incidence remains persistently high in clinical populations, where it is associated with inferior treatment outcomes, diminished psychological insight, and maladaptive coping mechanisms [12]. Neurobiological studies suggest that disruption in limbic and prefrontal circuits, along with altered neurotransmitter systems—especially those related to dopamine and serotonin—may be factors contributing to alexithymia [11,12]. Considering iron’s critical function in neurotransmitter systems and its impact on brain connections and white matter integrity, it is conceivable that iron deficiency may contribute to alexithymia via common pathophysiological mechanisms [5,11].

Moreover, although alexithymia is frequently considered a stable personality feature, recent data indicate that state-dependent factors, such as medical illnesses and nutritional inadequacies, may affect its manifestation over time [12]. This suggests that rectifying iron deficiency may have advantages for both physical health and emotional regulation, as well as overall mental well-being.

Women of reproductive age represent a critical demographic for examining these linkages. They have a heightened risk of iron insufficiency owing to variables such as menstruation, pregnancy, and lactation, which significantly enhance iron requirements [1,2]. This group simultaneously demonstrates increased susceptibility to mood and anxiety disorders, influenced by intricate biopsychosocial factors [13,14]. Examining the relationship between iron deficiency and emotional control in women is clinically significant and essential for public health, considering its possible cascade effects on family dynamics, child development, and overall population health.

Furthermore, alexithymia has been demonstrated to inhibit help-seeking behaviors and diminish compliance with medical treatments, potentially worsening chronic illnesses such as anemia [10,12]. Should iron deficiency correlate with heightened alexithymia levels, it could establish a detrimental cycle wherein emotional unawareness hampers self-care, prolongs diagnosis, and results in inadequate treatment outcomes. Understanding these interactions is crucial for formulating effective, patient-centered therapies that tackle both the medical and psychological aspects of anemia management. This integrated approach corresponds with modern holistic healthcare models that emphasize whole patient well-being over limited disease-centric management.

Although the theoretical feasibility of these links exists, practical studies directly investigating the relationship between IDA and alexithymia are few. Most current research has concentrated on general psychiatric symptoms, including sadness and anxiety, without evaluating the emotional processing challenges that may contribute to or exacerbate these disorders [7,8,10]. Furthermore, there is scant research investigating the correlation between iron status indicators—such as hemoglobin levels, ferritin concentrations, or total iron-binding capacity—and alexithymic traits [5,6]. Addressing this insufficiency is essential for cultivating a more thorough comprehension of iron deficiency’s effects on mental health.

The objective of this study was to comprehensively assess alexithymia, psychiatric symptoms, quality of life, and the prevalence of iron deficiency anemia among women aged 18–49 years, to evaluate their associations with laboratory indicators of iron status, and to compare these findings with those obtained from a healthy control group. This research utilized validated psychometric tools and structured psychiatric interviews to evaluate the prevalence of alexithymia in this population and investigate its correlations with specific biological indicators of iron status, as well as broader measures of psychological distress and well-being. This paper emphasizes the necessity of integrating emotional health evaluations into standard anemia management by promoting comprehensive, interdisciplinary strategies that combine biological and psychological care to enhance clinical results and quality of life for women.

## 2. Materials and Methods

This was a cross-sectional, case–control study designed to investigate the association between iron deficiency anemia (IDA) and alexithymia among women of reproductive age (years), while also examining symptoms of depression, anxiety, and quality of life. The case–control approach is widely employed in clinical research to explore associations between exposures and outcomes while minimizing confounding by using well-matched comparison groups [15,16,17].

### 2.1. Ethical Considerations

Ethical approval was obtained from the Firat University Non-Interventional Research Ethics Committee on 25 September 2023 (Reference No: E-50716828-100-375697). The study adhered to the principles of the 2013 edition of the Declaration of Helsinki. All participants provided written informed consent after receiving detailed information about the study’s purpose, procedures, potential risks, and benefits.

### 2.2. Study Setting and Period

Participants were recruited between September 2023 and February 2024 from Elazig Fethi Sekin City Hospital, a large tertiary referral center that serves a diverse regional population, enhancing the generalizability of the results.

G*Power statistical software (version 3.1.9.7) analysis was used to estimate a sample size. The calculation ensured 95% statistical power at a significance level (alpha error probability) of 0.05. To achieve sufficient statistical power, a total of 301 samples were included, with a projected required sample size of 289.

The hemoglobin concentration was measured using the cyanmethemoglobin method with an automatic hematology analyzer. A complete blood count (CBC) was performed using an automated hematology analyzer (UniCel DxH 800, Beckman Coulter, Inc., Miami, FL, USA). In the diagnosis of iron deficiency anemia in women, a hemoglobin level < 12 g/dL and serum ferritin ≤ 15 ng/mL were used as criteria. Serum ferritin (reference range: 15–200 ng/mL), serum iron (50–170 µg/dL), and total iron binding capacity (TIBC) (250–450 µg/dL) levels were detected using standard immunological analyses and colorimetric methods. Transferrin saturation, serum iron, and total iron-binding capacity (TIBC) values were used to conduct a more comprehensive analysis of iron status. Other blood measurements like mean corpuscular volume (MCV) and red cell distribution width (RDW) were taken to help confirm the diagnosis of iron deficiency anemia. All analyses were conducted in accordance with standard procedures and quality control principles in the hospital’s central laboratory.

**Figure 1:**
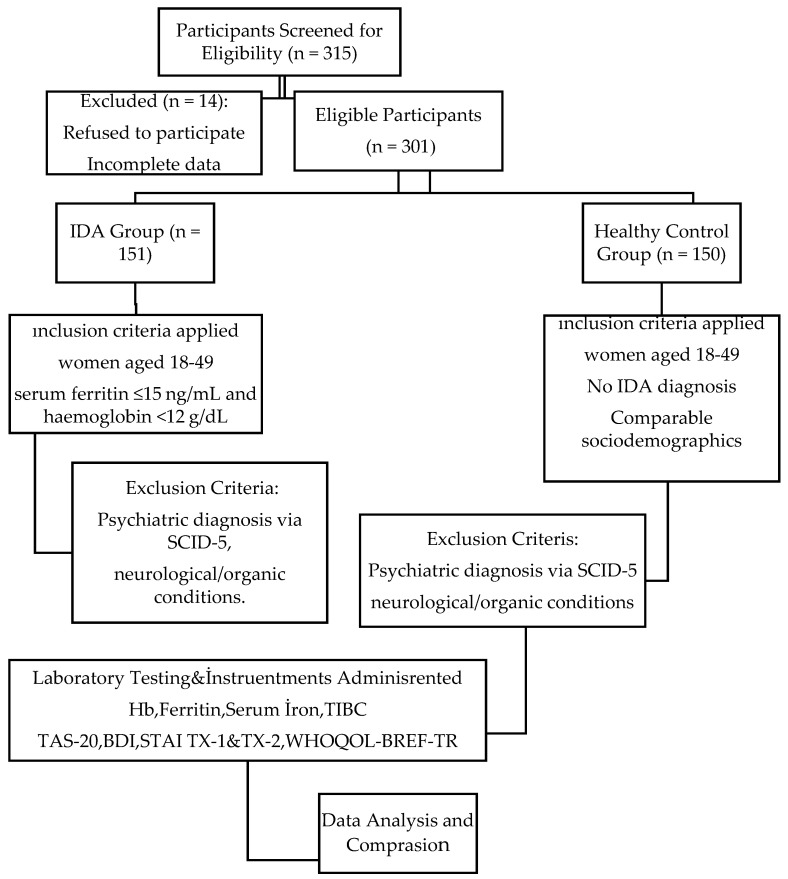
Flowchart of the design of study.

The overall framework and design of the study, along with the inclusion and exclusion criteria for participant selection, are summarized in Figure 1.

### 2.3. Sampling and Recruitment Procedures

We employed consecutive sampling, which is recommended in clinical research to reduce selection bias and improve external validity by ensuring that all eligible patients during the recruitment period are considered for inclusion [18]. Women diagnosed with IDA were consecutively approached during routine hospitalization or outpatient visits in the Internal Medicine Clinic. Eligible patients were identified from daily admission records and invited during routine clinical assessments. as summarized in the study flowchart (Figure 1).

Similarly, healthy controls were recruited consecutively from women attending the hospital’s annual health check-up service, ensuring they were comparable in terms of age and sociodemographic characteristics. During recruitment interviews, trained clinical staff provided detailed study information, answered participants’ questions, and obtained written informed consent from volunteers who met the eligibility criteria.

### 2.4. Inclusion and Exclusion Criteria

Inclusion criteria

- Female sex
- Age between 18 and 49 years
- Ability to provide informed consent

For the IDA group, diagnosis required meeting WHO-recommended laboratory criteria: serum ferritin ≤ 15 ng/mL and hemoglobin < 12 g/dL [19,20].

Exclusion criteria

- Any current psychiatric disorder diagnosed via structured clinical interview
- Intellectual disabilities impairing participation
- Organic or neurological conditions affecting cognitive function
- Anemia due to causes other than iron deficiency

### 2.5. Psychiatric Screening and Exclusion

To minimize confounding from psychiatric comorbidity, all participants underwent a structured clinical interview based on DSM-5 criteria (SCID-5), conducted by an experienced psychiatrist. These interviews lasted approximately 30 min and were performed in private clinical settings to ensure confidentiality and diagnostic precision. Participants with any current psychiatric diagnosis were excluded from the study. This approach aligns with recommended best practices for achieving sample homogeneity and reducing misclassification bias in psychiatric research [21,22].

### 2.6. Final Sample Size

Initially, 160 women with IDA and 155 healthy controls were approached. Following exclusions due to refusal (9 in IDA group) and incomplete data (5 in control group), the final sample consisted of 151 women with iron deficiency anemia and 150 healthy control participants.

### 2.7. Data Collection

After providing consent, participants completed a sociodemographic and clinical data form that recorded age, marital status, education, residence, household income, smoking and alcohol use, and medical history. Laboratory data on hemoglobin, ferritin, serum iron, and total iron-binding capacity were collected following standard hospital protocols.

Patients meeting the diagnostic criteria for iron deficiency anemia (hemoglobin < 12 g/dL, ferritin < 15 ng/mL) were treated with standard oral ferrous (Fe^2+^) formulations, providing 100 mg of elemental iron daily over a three-month period. This approach aligns with established clinical guidelines to effectively restore iron stores and correct anemia, with counselling provided on adherence and absorption-enhancing strategies.

### 2.8. Psychometric Instruments

All participants completed the following validated instruments:

- Beck Depression Inventory (BDI): assessing depressive symptom severity; Turkish version validated with Cronbach’s alpha = 0.78 [9,10].
- State-Trait Anxiety Inventory (STAI Forms TX-1 and TX-2): measuring state and trait anxiety; Turkish validation with Cronbach’s alpha = 0.80 and 0.82, respectively [23,24].
- Toronto Alexithymia Scale-20 (TAS-20): evaluating alexithymia levels; Turkish validation with Cronbach’s alpha = 0.80. Cut-off scores: ≤51 (non-alexithymia), 52–60 (possible alexithymia), and ≥61 (alexithymia) [11,12].
- WHOQOL-BREF-TR: the Turkish version of the WHO Quality of Life Scale Short Form [20,25].

### 2.9. Statistical Examination

The Statistical Package for the Social Sciences (SPSS) version 21.0 was used for the statistical analyses. The categorical variables are presented as frequencies and percentages. The continuous variables were assessed using Kolmogorov–Smirnov test and histograms to find out if their distributions were normal or not. The normally distributed numerical parameters were compared using student’s *t*-test in groups, while those with non-normal distributions were analyzed using Mann–Whitney U tests. The categorical variables were compared using chi-square or Fisher’s exact tests where appropriate. The strength of the relationship between the two variables was assessed using Spearman’s or Pearson’s correlation coefficients. A statistical difference was considered when *p*-value < 0.05. Univariate logistic regression analysis and multivariate logistic regression analysis with a stepwise approach were performed. Variables that remained significant (*p* < 0.05) in the multivariate model were considered as independent predictors of probable alexithymia or alexithymia. Hosmer–Lemeshow goodness of fit statistics were performed to assess model fit. Odds ratios (ORs) and 95% confidence intervals (CIs) were calculated for each predictor.

## 3. Results

A total of 301 female participants were included in the study, with 150 in the iron-deficiency anemia group and 151 in the control group. The median age was 34 (16–68). There were no significant differences in marital status, level of education, place of residence, as well as alcohol and cigarette consumption. However, the iron-deficiency anemia group was slightly older (36 years (IQR: 28–41) vs. 31 (IQR: 26–41)) and had higher household income level compared to the control group. Table 1 summarizes the baseline characteristics of patients.

With respect to laboratory test results and different scales used, hemoglobin, iron, ferritin levels, WHOQOL-BREF and STAI-1 score levels were lower, but total iron binding capacity and Toronto Alexithymia Scale score was higher in the iron-deficiency anemia group compared to the control group, as shown in Table 2.

When patients were categorized into two groups (without alexithymia vs. with likely alexithymia or alexithymia), 126 (42%) patients were identified as having probable alexithymia or alexithymia. No significant variations in age or demographic data were observed between the groups, with the exception of place of residence.

The prevalence of iron-deficiency anemia, Beck Depression Scale scores, and STAİ Form TX-2 scores were elevated in the likely alexithymia group compared to the non-alexithymia group, whereas WHOQOL-BREF-TR scores were diminished in the former group (Table 3).

To demonstrate the homogeneity of the IDA group regarding disease duration and treatment, the following table summarizes participant characteristics at baseline and the planned standard therapy (Table 4).

There are 150 patients with IDA. No significant differences were found in terms of age, marital status, education, residence, income, and alcohol and cigarette consumption. Moreover, there was no significant difference in the iron profile between the no alexithymia and probable alexithymia or alexithymia groups in patients with IDA.

The relationship between the Toronto Alexithymia Scale score and other numerical parameters is shown in Table 5. The Toronto Alexithymia Scale score positively correlated with total iron binding capacity, Beck Depression Scale, and STAI-2 score and negatively correlated with hemoglobin, Fe (iron), and WHOQOL-BREF score.

A binary logistic regression analysis was performed to detect the possible parameters that affect probable alexithymia or alexithymia. Multivariate analysis revealed that high total iron binding capacity (OR: 1.003, 95% CI: 1.000–1.006; *p* = 0.044), high STAI-2 score (OR: 1.098, 95% CI: 1.050–1.147; *p* < 0.001), and low WHOQOL-BREF score (OR: 0.966, 95% CI: 0.950–0.982; *p* < 0.001) were independently associated with probable alexithymia or alexithymia. The results of logistic regression analysis are summarized in Table 6.

Table 6 provides a summary of the logistic regression results. The following instruments were employed in the present study: the Beck Depression Scale, WHOQOL-BREF-TR, STAI Forms TX-1 and TX-2, and the Toronto Alexithymia Scale.

A sociodemographic and clinical data form, developed by the researchers, was utilized in alignment with information derived from clinical experience and vetted sources, tailored to the study’s objectives. The Beck Depression Scale (BDS) was created to assess the risk of depression, the intensity of depressive symptoms, and variations in severity among individuals. A validity and reliability assessment of the scale in Turkish was performed [14,26]. The Cronbach’s alpha reliability coefficient for the overall scale in this investigation was determined to be 0.78.

The Toronto Alexithymia Scale (TAS-20) is a self-assessment tool including 20 items designed to evaluate the degree of alexithymia. A validity and reliability evaluation of the scale in Turkish was performed [10,27]. This study established the cut-off values for alexithymia: a score of 51 or less indicated non-alexithymia, a score between 52 and 60 suggested potential alexithymia, and a score of 61 or above confirmed alexithymia. The Cronbach’s alpha reliability coefficient for the entire scale was determined to be 0.80. The State-Trait Anxiety Inventory (STAI Form TX-1, TX-2) was created by Spielberger. Validity and reliability research was undertaken in Turkey. The inventory comprises two sections and contains 40 products. STAI-1 evaluates state anxiety with items 1–20, while STAI-2 analyses trait anxiety using items 21–40. The average score on the scale ranges from 36 to 41 [13,28]. This study evaluated the Cronbach’s alpha reliability coefficient of the scale as 0.80 for STAI 1 and 0.82 for STAI 2. The Turkish version of the World Health Organization Quality of Life Scale Short Form (WHOQOL-BREF-TR) was established by the WHO, and a study on its validity and reliability in Turkish was performed [29,30].

In summary, these findings demonstrate significant disparities in sociodemographic profiles, hematological parameters, and psychological outcomes between groups. Elevated alexithymia, anxiety, and diminished quality of life in IDA patients underscore the critical importance of integrating psychological assessment and intervention into the standard clinical management of iron deficiency anemia to improve patient outcomes.

## 4. Discussion

This study contributes to the limited existing literature by comprehensively addressing the relationships between alexithymia levels, psychiatric symptoms, and quality of life in women with iron deficiency anemia (IDA). Our findings suggest that EIA may not only be a hematologic disorder, but also a systemic condition that can affect cognitive and emotional functioning. This perspective suggests the need for a more detailed understanding of the biological underpinnings of mental symptom clusters and declines in quality of life and may be an important basis for future research. This study revealed that women with iron deficiency anemia exhibited elevated levels of alexithymia, moderate anxiety, and diminished quality of life in comparison to healthy controls. The prevalence of iron deficiency anemia, depressive symptoms, and trait anxiety levels escalated in individuals who struggled to articulate their emotions, resulting in a diminished quality of life. The alexithymia levels in female patients with iron-deficient anemia were correlated with elevated iron-binding capacity, heightened anxiety levels, and diminished quality of life.

Emotional behaviors are influenced by iron levels in the brain, especially in cases of iron shortage. The correlation between hunger and depression has been proposed in women of reproductive age. Lee et al. demonstrated that patients with iron-deficient anemia exhibited elevated anxiety levels and a high prevalence of depression [6]. Another study of 100 cases and 100 controls showed an association between iron deficiency anemia and depressive disorder, and the severity of depressive disorder symptoms increased with the degree of anemia [31]. A separate investigation indicated that the treatment of iron deficiency alleviated feelings of anxiety and sadness in patients [7]. Furthermore, diminished hemoglobin and plasma ferritin levels have been correlated with depression [32,33]. Iron deficiency impairs the mineral structure as well as the function of some enzymes involved in neurotransmitter synthesis, including serotonin, dopamine, and norepinephrine [34]. Another study has shown that impaired emotional behavior is associated with iron deficiency through altered dopamine metabolism [35,36]. All these possible mechanisms may explain why iron deficiency may increase the risk of psychiatric disorders. Our investigation revealed no disparity in depressive symptoms between women with iron deficiency and healthy women; however, those with anemia exhibited moderate levels of state anxiety. In this context, our research diverges from the existing literature.

Studies indicate that iron deficiency may affect alexithymia via intricate neurodevelopmental and neurobiological processes. In addition to its function in neurotransmitter production, iron is crucial for myelination and synaptic plasticity, especially in brain areas associated with emotional processing, including the prefrontal cortex, anterior cingulate cortex, and insula [37,38]. Deficiencies at crucial developmental phases might lead to enduring modifications in dopaminergic and glutamatergic signaling, thereby exposing individuals to emotional dysregulation and diminished interoceptive awareness, a fundamental characteristic of alexithymia [39]. Animal models indicate that prenatal iron deficit leads to enduring decreases in dopamine receptor density and modified stress responsivity, corroborating a developmental programming theory [40]. Moreover, data indicate that iron deficiency correlates with persistent low-grade inflammation and modified hypothalamic–pituitary–adrenal (HPA) axis function [41]. These alterations can interfere with emotional regulation networks and may partially elucidate the concomitant anxiety and depression symptoms commonly found in persons with alexithymia. Recent neuroimaging research reveals diminished functional connectivity in emotion-related networks in people with iron-deficient anemia and high alexithymia scores [42]. Intervention studies are increasingly focusing on this junction. Initial studies indicate that iron repletion may enhance both hematologic parameters and mood, as well as cognitive performance, while its impact on emotional awareness is still inadequately investigated [43]. This highlights the necessity for comprehensive treatment strategies that merge dietary rehabilitation with psychological therapies aimed at addressing emotional processing deficiencies. Future study should utilize longitudinal designs to ascertain if early intervention for iron insufficiency might avert the emergence of alexithymia features. Furthermore, precision medicine strategies that integrate iron status biomarkers may assist in identifying at-risk individuals who would gain the most from customized interventions. These discoveries highlight that alexithymia associated with iron deficiency is not simply a comorbidity but may indicate common pathophysiological causes that require an interdisciplinary treatment focus.

Anemia diminishes patients’ quality of life, which deteriorates more as hemoglobin levels decline [44]. Iron supplementation enhances the quality of life for those with anemia [45]. Prior research on individuals with anemia indicates that their quality of life diminishes across nearly all domains, encompassing emotional well-being and overall health perception, with the most significant decline observed in aspects connected to physical activity. Furthermore, a decline in hemoglobin levels correlates with a deterioration in quality of life [46,47]. Our study indicated that the quality of life for women with iron-deficient anemia was diminished. In this regard, our research aligns with other studies. Iron deficiency anemia is a multifactorial condition that has a multidimensional negative impact on quality of life in women. Decreased hemoglobin levels lead to insufficient oxygen transport to tissues, causing symptoms such as fatigue, physical limitations, and exercise intolerance [48]. Iron can also affect mood due to the above-mentioned conditions. In addition, iron deficiency anemia, impaired sleep quality, and restless legs syndrome reduce daily functioning [49,50]. This physiological and psychological burden can lead to poor social relationships and impaired emotional functioning, significantly reducing quality of life.

Our study’s most significant conclusion is that alexithymia scores are elevated in patients with iron-deficient anemia. Alexithymia, characterized by the inability to identify and articulate emotions, arises from the influence of dopaminergic and glutamatergic systems in the brain [51]. Alexithymia is known to be prevalent, particularly in neurological diseases characterized by dopaminergic dysfunction [11]. The alexithymia levels in patients with restless legs syndrome attributed to iron insufficiency were similarly shown to be elevated [12]. The iron element, integral to the production and operation of neurotransmitters, may induce alexithymia symptoms via the potential dopaminergic system. Moreover, individuals with alexithymia may exhibit symptoms of malnutrition and eating disorders, such as food intake restriction, binge eating, and/or vomiting [52]. This indicates that this circumstance may induce iron shortage in individuals with alexithymia, whereas those with iron deficiency anemia may exhibit alexithymia, corroborating our findings. Alexithymia escalates with a reduction in ferritin levels [53]. Our study revealed a significant prevalence of iron deficiency anemia among individuals with elevated alexithymia scores. A positive link was identified between alexithymia and iron binding capacity, whereas a negative correlation was detected with hemoglobin and iron levels. The relationship between iron deficiency anemia (IDA) and alexithymia can be explained through the neurobiological functions of iron and its role in emotional processing. In fact, iron is a cofactor that plays a critical role in the function of many neurotransmitters that function extensively in brain regions associated with emotional awareness, identification of internal states, and social cognition, particularly in the prefrontal cortex, anterior cingulate cortex, and insula. Iron deficiency can lead to dysfunction in neurotransmitters in these regions, impairing the perception, identification, and verbalization of emotions, representing functional deficits that underlie alexithymia. Chronic hypoxia, mitochondrial dysfunction, and neuroinflammation associated with iron deficiency can impair the integrity of neurocircuits mediating emotional processing. Thus, it may make it difficult for the individual to distinguish internal emotional states and produce appropriate cognitive-linguistic responses to these states, which may lead to alexithymia symptoms. All these factors suggest that iron deficiency anemia may constitute a biological risk factor in the development of alexithymia. It was also established that an increase in alexithymia correlated with heightened depressive symptoms and trait anxiety levels, while quality of life diminished. In this context, our data align with the existing literature for comparable parameters assessed in other disorders [54,55]. Consequently, individuals with alexithymia are perceived to have heightened anxiety and depression, perhaps leading to a diminished quality of life. Moreover, it may be asserted that elevated iron binding capacity, diminished quality of life, and heightened trait anxiety are independently linked components contributing to the onset of alexithymia.

### Limitation

Some limitations of this study should be considered. The cross-sectional design limits inferences about causal relationships. The use of self-report-based scales increases the risk of response bias. Finally, the fact that biological markers (e.g., ferritin, hepcidin) were not assessed in the study prevented the elucidation of potential biological mechanisms between psychiatric symptoms and iron metabolism. One of our strengths is that the sample group comprised females, and there is a lack of prior studies assessing the link among anxiety, depression, quality of life, and alexithymia in patients with iron deficiency anemia.

## 5. Conclusions

This study examined the relationship among alexithymia, psychiatric symptoms and quality of life in women with iron deficiency anemia (IDA) in a holistic manner and revealed that IDA is a systemic condition that includes not only hematological but also psychological and functional dimensions. The findings emphasize the effects of alexithymia on both psychiatric symptom burden and quality of life and suggest that emotional processing difficulties may be a determinant in the clinical course of IDA. The study contributes to the expansion of psychosomatic models by drawing attention to potential links between the biological basis of alexithymia and iron metabolism. It also suggests that individuals with iron deficiency anemia should be included in psychiatric evaluation processes, and emotional awareness-based interventions should be integrated into treatment. However, the cross-sectional design limits causal inferences; longitudinal and biological data-supported approaches are recommended for future studies. In addition, the development of holistic theoretical models to explain the interactions between alexithymia and physiological parameters will make the understanding in this field even more valuable. Future studies with larger sample sizes are warranted to further investigate the relationship between iron-deficiency anemia and alexithymia, ideally incorporating analyses of underlying neurobiological mechanisms, particularly those involving neurotransmitters. A clearer understanding of this association could facilitate non-invasive diagnostic approaches to anemia. Moreover, the evaluation and management of emotional symptoms such as sadness and anxiety during treatment and follow-up may contribute to improved quality of life in affected patients.

## Figures and Tables

**Table 1:** Baseline characteristics of participants.

	Control (n = 151)	Iron-Deficiency Anemia Group (n = 150)	*p*-Value
Sex			
n (%)	Female 151 (100)	Female 150 (100)	
Marital Status			0.131
Single or widowed, n (%)	62 (41.1)	49 (32.7)	
Married, n (%)	89 (58.9)	101 (67.3)	
Age, median (IQR)	31 (26–41)	36 (28–41)	0.048
Level of Education			0.803
Illiterate, n (%)	1 (0.7)	1 (0.7)	
Primary school, n (%)	15 (9.9)	15 (10)	
High school, n (%)	33 (21.9)	40 (26.7)	
University, n (%)	102 (67.5)	94 (62.7)	
Place of residence			0.767
Rural area, n (%)	6 (4)	7 (4.7)	
Urban area, n (%)	145 (96)	143 (95.3)	
Household income level			0.026
Low, n (%)	28 (18.5) *	14 (9.3) *	
Medium, n (%)	111 (73.5) *	129 (86) *	
High, n (%)	12 (8)	7 (4.7)	
Alcohol consumption			0.564
No, n (%)	144 (95.4)	145 (96.7)	
Yes, n (%)	7 (4.6)	5 (3.3)	
Smoking			0.108
No, n (%)	126 (83.4)	114 (76)	
Yes, n (%)	25 (16.6)	36 (24)	
History of psychiatric illness			0.501
No, n (%)	148 (98)	145 (96.7)	
Yes, n (%)	3 (2)	5 (3.3)	
Family history of psychiatric illness			0.501
No, n (%)	148 (98)	145 (96.7)	
Yes, n (%)	3 (2)	5 (3.3)	
Suicide attempt			0.996
No, n (%)	150 (99.3)	149 (99.3)	
Yes, n (%)	1 (0.7)	1 (0.7)	
History of iron-deficiency anemia treatment			0.014
No, n (%)	68 (45)	47 (31.3)	
Yes, n (%)	83 (55)	103 (68.7)	

* *p* values significantly different between groups in each row. Categorical variables are presented as n (%). Normally distributed variables are presented as mean ± standard deviation. Variables with a skewed distribution are presented as median (IQR (interquartile range)).

**Table 2:** Laboratory and psychiatric scales results of the participants.

Variable	Control (n = 151)	IDA (n = 150)	*p*
Hemoglobin, g/dL median (IQR)	13.7 (13–14)	10.1 (9–11)	<0.001
Iron (Fe), µg/dL median (IQR)	43 (30–63)	25 (17–32)	<0.001
Ferritin, ng/mL median (IQR)	15 (11–27)	7 (4–10)	<0.001
Total iron binding capacity, µg/dL median (IQR)	289 (254–328)	369 (299–401)	<0.001
Beck Depression Scale, median (IQR)	9 (4–15)	9 (4–14)	0.788
Toronto Alexithymia Scale, mean ± SD	48.4 ± 9.4	52 ± 9.3	0.001
WHOQOL-BREF-TR, median (IQR)	97 (87–104)	87 (77–98)	<0.001
STAI Form TX-1, median (IQR)	42 (38–47)	40 (36–45)	0.007
STAI Form TX-2, median (IQR)	44 (40–49)	46 (41–49)	0.077

Categorical variables are presented as n (%). Normally distributed variables are presented as mean ± standard deviation. Variables with a skewed distribution are presented as median (IQR (interquartile range)).

**Table 3:** Characteristics of participants between the no alexithymia and probable alexithymia or alexithymia groups.

Variable	No Alexithymia (n = 175)	Probable Alexithymia or Alexithymia (n = 126)	*p*
Age, years median (IQR)	34 (26–41)	35 (28–42)	0.333
Marital Status			0.551
Single or widowed, n (%)	67 (38.3)	44 (34.9)	
Married, n (%)	108 (61.7)	82 (65.1)	
Level of Education			0.230
Illiterate, n (%)	14 (8)	16 (12.7)	
Primary school, n (%)	47 (26.9)	26 (20.6)	
High school, n (%)	112 (64)	84 (66.7)	
University, n (%)	2 (1.1)	0 (0)	
Place of residence			0.041
Rural area, n (%)	4 (2.3)	9 (7.1)	
Urban area, n (%)	171 (97.7)	117 (92.9)	
Household income level			0.083
Low, n (%)	31 (17.7)	11 (8.7)	
Medium, n (%)	133 (76)	107 (84.9)	
High, n (%)	11 (6.3)	8 (6.3)	
Alcohol consumption			0.989
No, n (%)	168 (96)	121 (96)	
Yes, n (%)	7 (4)	5 (4)	
Smoking			0.876
No, n (%)	139 (79.4)	101 (80.2)	
Yes, n (%)	36 (20.6)	25 (19.8)	
Iron-deficiency Anemia			0.002
No, n (%)	101 (57.7)	50 (39.7)	
Yes, n (%)	74 (42.3)	76 (60.3)	
Hemoglobin, g/dL median (IQR)	13 (10.7–14)	11.4 (10–13.3)	0.017
Iron (Fe), µg/dL median (IQR)	34 (24–50)	29 (18–45)	0.062
Ferritin, ng/mL median (IQR)	11 (7–20)	9 (6–18)	0.288
Total iron binding capacity, µg/dL median (IQR)	302 (275–379)	334 (280–389)	0.084
Beck Depression Scale, median (IQR)	7 (3–13)	10 (7–17)	<0.001
WHOQOL-BREF, median (IQR)	98 (87–108)	86 (78–94)	<0.001
STAI-1, median (IQR)	42 (38–46)	41 (37–46)	0.443
STAI-2, median (IQR)	44 (40–47)	46 (42–51)	<0.001

Categorical variables are presented as n (%). Normally distributed variables are presented as mean ± standard deviation, and skewed variables are presented as median (min–max). min: minimum, max: maximum.

**Table 4:** Characteristics of participants with IDA.

	No Alexithymia (n = 74)	Probable Alexithymia or Alexithymia (n = 76)	*p*
Age, years, median (min–max)	36 (27–41)	36 (29–42)	0.955
Marital Status			0.602
Single or widowed, n (%)	26 (35.1)	23 (30.3)	
Married, n (%)	48 (64.9)	53 (69.7)	
Level of Education			0.202
Illiterate, n (%)	1 (1.4)	0 (0)	
Primary school, n (%)	5 (6.8)	10 (13.2)	
High school, n (%)	24 (32.4)	16 (21.1)	
University, n (%)	44 (59.4)	50 (65.7)	
Place of residence			0.442
Rural area, n (%)	2 (2.7)	5 (6.6)	
Urban area, n (%)	72(97.3)	71 (93.4)	
Household income level			0.815
Low, n (%)	6 (8.1)	8 (10.5)	
Medium, n (%)	65 (87.8)	64 (84.2)	
High, n (%)	3 (4.1)	4 (5.3)	
Alcohol consumption			0.671
No, n (%)	72 (97.3)	73 (96.1)	
Yes, n (%)	2 (2.7)	3 (3.9)	
Smoking			0.501
No, n (%)	58 (78.4)	56 (73.7)	
Yes, n (%)	16 (21.6)	20 (26.3)	
Hemoglobin, g/dL median (IQR)	10 (9–11)	10.1 (9.2–11.1)	0.454
Iron (Fe), µg/dL median (IQR)	26 (17–34)	24.5 (17–31)	0.855
Ferritin, ng/mL median (IQR)	7 (4–10)	7 (4–11)	0.564
Total iron binding capacity, µg/dL median (IQR)	350 (299–399)	379 (299–403)	0.472

**Table 5:** Correlation between Toronto Alexithymia Scale score and other numerical parameters.

		Toronto Alexithymia Scale
Age	r	0.009
	*p*-value	0.873
Hb	r	−0.175
	*p*-value	0.002
Fe	r	−0.164
	*p*-value	0.004
Ferritin	r	−0.069
	*p*-value	0.235
Total iron binding capacity	r	0.117
	*p*-value	0.042
Beck Depression Scale	r	0.302
	*p*-value	<0.001
WHOQOL-BREF	r	−0.431
	*p*-value	<0.001
STAI-1	r	−0.095
	*p*-value	0.102
STAI-2	r	0.169
	*p*-value	0.003

*p* < 0.05; Pearson correlation was used.

**Table 6:** Independent predictors of probable alexithymia or alexithymia.

Risk Factors	Adjusted OR (95% CI)	*p*-Value
Total iron binding capacity	1.003 (1.000–1.006)	0.044
Beck Depression Scale	1.032 (0.998–1.068)	0.068
WHOQOL-BREF-TR	0.966 (0.950–0.982)	<0.001
STAI Form TX-2	1.098 (1.050–1.147)	<0.001

95% CI, 95% confidence interval. The *p* value of the Hosmer–Lemeshow test was 0.059. The following factors were entered into the multivariate logistic regression analysis: level of education, household income level, place of residence, iron-deficiency anemia, Beck Depression Scale, WHOQOL-BREF, STAI-2, iron (Fe), and total iron binding capacity.

## Data Availability

The authors affirm that the data underpinning the findings of this investigation are accessible within the article and additional materials.
